# Pathological Causes of Cyanosis

**Published:** 1844-03

**Authors:** 


### Selections from American and Foreign Journals.

---

*Pathological Causes of Cyanosis.*—Dr. Craigie has given, in the *Edinburgh Medical and Surgical Journal*, for October last, some very interesting observations on the pathological causes of cyanosis.

"It has been usually supposed," he remarks, "that the open state of the *foramen ovale*, whether direct or oblique, is a primary lesion of the heart, and is pernicious to the patient, in allowing the free intermixture of the blood of the right chambers of the heart with that of the left. Within certain limits this idea is well-founded; and in a certain number of cases, the open state of the *foramen ovale* tends to impede nutrition and to abridge the duration of life. I am, nevertheless, satisfied, both from the facts of the case now related, and several others, of three circumstances; *first*, that the open state of the *foramen ovale* is rarely a primary and solitary lesion; *secondly*, that when it is a solitary lesion it is not injurious, and the venous blood of the right auricle is not thereby necessarily mixed with the arterial blood of the left auricle; and, *thirdly*, that in opposition to what has been hitherto usually taught, the open state of the *foramen ovale* is in a large proportion of cases the means of prolonging life.

"It is unnecessary for me to enter into any formal defence the latter conclusion, which, however paradoxical it may seem, and however opposed to the usually received dogmas, flows almost directly from the facts which may be traced in every case of open *foramen ovale*. That, in short, it is not the primary lesion. From the phenomena of the cases recorded, on the contrary, and from the frequency of the arctated or contracted state of the pulmonary artery, it must be inferred that the obstructed state of that artery is the primary lesion, and determines not only the open state of the *foramen ovale*, but the hypertrophy of the right ventricle. This is the result, whether the pulmonary artery is only greatly narrowed in calibre, or terminates in a *cul de sac*, or is obstructed by a membranous partition formed by the coalition of the semilunar valves.

"The effect of such an impediment is manifest. The blood cannot pass into the pulmonary artery with the requisite freedom and facility. The result is over-distension, *first* of the right ventricle, and excessive labor of its muscular apparatus; *secondly*, of the right auricle, and excessive labor of its muscular apparatus, with extreme dilatation of its membranous portion; *thirdly*, over-distension and congestion of the whole venous system all over the body. The lungs, meanwhile, receive little or no blood, and, consequently, the blood is not duly aerated or supplied with oxygen, and cleared of carbon and carbonic acid. This is doubtless an evil and a great one. But Bichat has obscurely suggested, and Dr. Williams and Dr. Kay have clearly shown, that dark-colored blood, or that which is venous, is adequate to maintain vital action. It is, indeed, a less evil, and more tolerable than total obstruction in any of the large vessels, and especially in a vessel like the pulmonary artery. Everything that we now know of these cases shows that the obstruction to the circulation through the pulmonary artery must be the main cause of the short and transitory existence of persons laboring under this severe lesion; and that the open state of the *foramen ovale*, instead of being, as William Hunter and other authors imagined, a cause of death, furnishes the only means by which life can be prolonged, while a function so important as that of the circulation through the lungs is impeded.

"I am further entitled to infer from various facts in the history of the development of the ovum, that the obstructed, or it may be, the undeveloped state of the pulmonary artery, is the anatomical cause of the perforated septum, and of the origin of the aorta from the two ventricles when that malformation is observed.

"It would lead to some curious and interesting results to inquire by what means the impeded function of the lungs is in these cases compensated; for that it is compensated by the action of the skin and other membranes, can scarcely be doubted. But this would lead me into a field too extensive for consideration at the present time."

